# Pyrimidinergic P2Y1-Like Nucleotide Receptors Are Functional in Rat Conjunctival Goblet Cells

**DOI:** 10.1167/iovs.66.1.46

**Published:** 2025-01-21

**Authors:** Ketil A. Fjærvoll, Haakon K. Fjærvoll, Menglu Yang, Jeffrey Bair, Tor P. Utheim, Darlene A. Dartt

**Affiliations:** 1 Schepens Eye Research Institute, Massachusetts Eye and Ear Infirmary, Department of Ophthalmology, Harvard Medical School, Boston, Massachusetts, United States; 2 Division of Head, Neck and Reconstructive Surgery, Institute of Clinical Medicine, Faculty of Medicine, University of Oslo, Oslo, Norway; 3 Medical Student Research Program, Institute of Clinical Medicine, Faculty of Medicine, University of Oslo, Oslo, Norway; 4 Department of Medical Biochemistry, Oslo University Hospital, Oslo, Norway

**Keywords:** calcium, conjunctiva, goblet cells, ocular surface, purinergic receptors, UTP

## Abstract

**Purpose:**

To investigate the presence of uridine-5′-triphosphate (UTP)-activated P2Y1-like nucleotide receptors (P2Y_2_R, P2Y_4_R, and P2Y_6_R) in conjunctival goblet cells (CGCs) and determine if they increase intracellular Ca^2+^ concentration ([Ca^2+^]*_i_*) and induce mucin secretion.

**Methods:**

Adult, male rat conjunctiva was used for culture of CGCs. To investigate the expression of P2YRs, mRNA was extracted from CGCs and used for reverse transcription PCR (RT-PCR) with commercially obtained primers specific to P2Y_2_R, P2Y_4_R, and P2Y_6_R. Immunofluorescence (IF) and western blot (WB) analyses were performed using first-passage CGCs and stained with antibodies specific to each P2YR. Furthermore, CGCs were incubated with fura-2/AM, and [Ca^2+^]*_i_* was measured after stimulation with the P2YR selective agonists UTP, uridine 5′-diphosphate (UDP), or UDP–glucose and agonists specific to P2Y_2_R (MRS 2768), P2Y_4_R (MRS 4062), and P2Y_6_R (MRS 2693). [Ca^2+^]*_i_* measurements after P2Y_2_R and P2Y_6_R siRNA treatment were performed. Mucin secretion was measured after stimulation of P2Y_2_R, P2Y_4_R, and P2Y_6_R.

**Results:**

mRNA for all pyrimidinergic P2Y_1_-like receptors was found as single bands of expected base pair number with RT-PCR. The presence of these P2YRs was confirmed with IF microscopy and WB analysis. UTP and UDP elicited concentration-dependent increases in [Ca^2+^]*_i_*. The receptor-specific agonists and UDP–glucose increased [Ca^2+^]*_i_*, although these responses were substantially lower than those elicited by UTP and UDP at 10^–4^ M and 10^–3^ M and did not show similar dose dependency. P2Y_2_R- and P2Y_6_R-depleted CGCs responded with reduced peak [Ca^2+^]*_i_*. UTP, MRS 2768 (P2Y_2_R), and UDP each stimulated mucin secretion from CGCs.

**Conclusions:**

P2Y_2_R, P2Y_4_R, and P2Y_6_R are present and functional in rat CGCs and may represent novel therapeutic targets for dry eye treatment and other types of ocular surface disease.

Dry eye disease (DED) is a highly prevalent, chronic, multifactorial disease with loss of functioning of the tear film that overspreads the cornea and conjunctiva.^1^^,^^2^ Despite the economic burden and aggravating symptoms of DED, few effective treatments are available.^1^^,^^3^^–^^5^

The conjunctival epithelium secretes water, electrolytes, and mucins into the tear film that collectively contribute to tear film stability, ocular surface protection, and maintenance of clear vision.^1^^,^^6^ Conjunctival goblet cells (CGCs) contain granules filled with mucins, predominantly mucin 5AC antibody (MUC5AC). MUC5AC-containing secretory vesicles in CGCs are important because MUC5AC is a high-molecular-weight, gel-forming mucin that provides lubrication to the eye and a scaffold for the mucous layer of the tear film.^1^^,^^6^ Normally, these vesicles are secreted by compound exocytosis upon activation of a parasympathetic neural reflex arc.^7^ However, compound exocytosis can also be regulated by inflammatory mediators which alter mucous secretion in those affected.^6^^,^^8^ Goblet cell loss and MUC5AC deficiency are observed in patients with DED.^9^

Purinergic signaling plays a prominent role in secretion in epithelial tissues.^10^ It is initiated through activation of purinergic receptors by inflammatory modulators such as adenosine, nucleotides, nucleotide sugars, and nucleotide analogs. The two types of purinergic receptors are the purinergic type 1 (P1) receptor, known as the adenosine receptor, and the purinergic type 2 (P2) receptor.^10^^,^^11^ P2 receptors are composed of two families of receptors. The first family is the ionotropic P2X receptor family, which includes seven different subtypes, P2X_1_ through P2X_7_. The second family is comprised of P2Y receptors and includes eight seven-membrane-spanning G protein–coupled receptors (P2Y_1_R, P2Y_2_R, P2Y_4_R, P2Y_6_R, P2Y_11_R, P2Y_12_R, P2Y_13_R, and P2Y_14_R). The first group of P2YRs (P2Y_1_R, P2Y_2_R, P2Y_4_R, P2Y_6_R, and P2Y_11_R), referred to as P2Y1-like nucleotide receptors, is mainly coupled to G_q_ proteins that activate phospholipase C. The second group (P2Y_12_R, P2Y_13_R, and P2Y_14_R), denoted P2Y12-like nucleotide receptors, is coupled to G_i_ proteins that inhibit adenylate cyclase. P2Y_11_R is reportedly absent in the murine genome.^10^^,^^12^ P2Y_1_R, P2Y_12_R, and P2Y_13_R are activated by the purine nucleotide adenosine diphosphate (ADP) and are not addressed here.^13^ Among the P2Y1-like nucleotide receptors, P2Y_2_R, P2Y_4_R, and P2Y_6_R are activated by the pyrimidine nucleotide uridine-5′-triphosphate (UTP).^13^^,^^14^

The corneal and conjunctival epithelia have a protective function and a location that exposes them to a wide variety of nucleotide-releasing stimuli.^10^^,^^15^ In addition, nucleotides can likely be released from nerves innervating the ocular surface.^15^ The purinergic receptors may therefore constitute an important family of receptors in the conjunctival epithelium, as these receptors likely play a significant role in the induction of mucin secretion. In fact, a drug against DED that targets the UTP-sensitive P2Y_2_R in CGCs to induce mucin secretion was developed but is reported to have suboptimal effects.^16^^,^^17^ Thus, there is room for further development and optimization of P2YR-targeting therapies for ocular surface pathologies.

P2Y_2_R is the only receptor of the P2Y1-like family of purinergic receptors that has been widely investigated in conjunctival tissue.^18^^–^^20^ The two other pyrimidinergic P2YRs in the P2Y1-like family that are activated by uridine nucleotides (P2Y_4_R and P2Y_6_R) could be regulators of mucin secretion in the CGCs. These receptors might function in both health and disease to maintain a clear cornea and protect vision. Recently developed agonists specific to each of the three UTP-activated P2Y1-like nucleotide receptors make it possible to study the receptors separately. Thus, in the present study, we examined the presence of P2Y_2_R, P2Y_4_R, and P2Y_6_R in rat CGCs and determined whether their activation stimulates CGC mucin secretion through elevation of intracellular Ca^2+^ levels.

## Materials and Methods

### Materials

Roswell Park Memorial Institute (RPMI) 1640 cell culture medium, penicillin/streptomycin, and l-glutamine were purchased from Lonza (Basel, Switzerland), and fetal bovine serum (FBS) was purchased from Bio-Techne (Minneapolis, MN, USA). *Ulex europaeus* agglutinin I (UEA-1), UTP trisodium salt hydrate, uridine 5′-diphosphate (UDP) disodium salt hydrate, pluronic acid F127, and sulfinpyrazone were obtained from Sigma-Aldrich (St. Louis, MO, USA). P2YR antibodies and control peptides were purchased from Alomone Laboratories (Jerusalem, Israel): P2Y_2_R, APR-010; P2Y_4_R, APR-006; and P2Y_6_R, APR-106. Cytokeratin-7 (CK7) antibody was purchased from Santa Cruz Biotechnology (Dallas, TX, USA), and Cy2 and Cy3 antibodies were purchased from Jackson ImmunoResearch Laboratories (West Grove, PA, USA). MRS 2365, MRS 2768, MRS 4062, and MRS 2693 were purchased from Tocris Bioscience (Bristol, UK). Fura-2 acetoxymethyl ester (fura-2/AM) and 1,2-*bis*(*o*-aminophenoxy)ethane-*N*,*N*,*N*′,*N*′-tetraacetic acid tetra(acetoxymethyl ester) (BAPTA/AM) were purchased from Life Technologies (Carlsbad, CA, USA). All primers were purchased from Integrated DNA Technologies (Coralville, IA, USA).

### Animals

All animal work conformed to the ARVO Statement for the Use of Animals in Ophthalmic and Vision Research. Male Sprague Dawley rats, 4 to 8 weeks old and weighing between 125 and 150 g, were fed ad libitum and obtained from Taconic Biosciences (Germantown, NY, USA). The rats were anesthetized with CO_2_ for 5 minutes and decapitated before the bulbar and forniceal conjunctiva were removed from both eyes. All experiments were approved by the Schepens Eye Research Institute Animal Care and Use Committee. Data were collected at Schepens Eye Research Institute, and derived data are available from the corresponding author upon reasonable request.

### Cell Culture

Goblet cells from rat conjunctiva were grown from explants in organ culture as described previously.^21^^,^^22^ The rat conjunctiva was dissected, and pieces of minced tissue were placed in six-well plates with 0.5 mL RPMI 1640 medium supplemented with 10% FBS, 2-mM l-glutamine, and 100 mg/mL penicillin–streptomycin. The RPMI 1640 medium was changed every second day. First-passage CGCs were used in all of the experiments. To ensure that goblet cells predominated, the identity of cultured cells was periodically checked via fluorescence microscopy staining with goblet cell–specific CK-7 and/or with the lectin UEA-1 to detect goblet cell secretory product.

### Reverse Transcription Polymerase Chain Reaction

Except for study specific modifications, reverse transcription PCR (RT-PCR) was performed as previously reported.^23^ Cultured CGCs were homogenized using QIAzol Lysis Reagent and total RNA was isolated according to the Quick-Start Protocol for the miRNeasy Mini Kit (QIAGEN, Hilden, Germany). Total RNA was treated with DNAse and purified as specified in the user guide for the TURBO DNA-free Kit from Invitrogen (Thermo Fisher Scientific, Waltham, MA, USA). The quality of RNA was assured, and concentrations were measured with a NanoDrop 2000c Spectrophotometer (Thermo Fisher Scientific). Three micrograms of purified total RNA were applied for complementary DNA (cDNA) synthesis using the Invitrogen SuperScript III First-Strand Synthesis System for RT-PCR (Thermo Fisher Scientific). The cDNA was amplified by PCR using primers specific to rat P2YRs and the JumpStart REDTaq ReadyMix PCR Reaction Mix (Sigma-Aldrich) in a thermal cycler (Master Cycler; Eppendorf, Hamburg, Germany). Samples with no cDNA served as the negative control, and the presence of β-actin served as the positive control. P2YR primer sequences (Supplementary Table S1) were derived from previously published articles^24^^–^^26^ and cross-checked using the Primer-BLAST function in the National Center for Biotechnology Information (NCBI) database. The following procedure was used: 7 minutes at 95°C; 40 cycles of 30 seconds at 94°C, 30 seconds at 60°C, and 60 seconds at 72°C; and a final countdown at 72°C for 5 minutes. Then, 10 µL PCR product and 1 µL 100-bp ladder with 5 µL 6× loading dye (Thermo Fisher Scientific) were separated on a 1% agarose gel (Tris-borate-EDTA [TBE] buffer) containing 5 µL of 10,000 GelRed (Biotium, Fremont, CA, USA) before visualization in a Gel Doc XR+ Gel Documentation System (Bio-Rad Laboratories, Hercules, CA, USA).

### Western Blot Analysis

Western blot (WB) analysis was conducted as described in previous publications,^23^ but with project-specific adjustments. Cultured CGCs were scraped on ice in radioimmunoprecipitation assay (RIPA) buffer containing 10-mM Tris HCl (pH 7.4), 150-mM NaCl, 1% deoxycholic acid, 1% Triton X-100, 0.1% sodium dodecyl sulfate (SDS), and 1-mM EDTA with protease inhibitors (phenylmethylsulfonyl fluoride 100 µL/mL, aprotinin 30 µL/mL) and 100-nM sodium orthovanadate. When homogenized, cells were sonicated before centrifugation at 2000*g* for 10 minutes at 4°C. Supernatant was isolated before the addition of 125 µL of 4× protein loading buffer (LI-COR, Lincoln, NE, USA) per 500 µL of supernatant. Proteins in the supernatant were separated by SDS–polyacrylamide gel electrophoresis on a 12% gel and transferred onto nitrocellulose membranes. The membranes were blocked overnight at 4°C in 5% nonfat dried milk in buffer containing 10-mM Tris HCl (pH 8.0), 150-mM NaCl, and 0.05% Tween 20 and then incubated with primary antibodies targeting P2YRs at a 1:200 dilution for 72 hours at 4°C followed by a 1-hour incubation with IRDye 680RD (LI-COR) (1:15,000 dilution). β-Actin and pre-incubation with control peptide for anti-P2Y_2_R served as controls. Immunoreactive bands were visualized with the Odyssey Classic Imager (LI-COR).

### Immunofluorescence Microscopy

In addition to minor modifications, immunofluorescence (IF) microscopy was performed as previously described.^23^ First-passage CGCs were grown on glass coverslips and fixed with 4% paraformaldehyde diluted in phosphate-buffered saline (PBS; 145-mM NaCl, 7.3-mM Na_2_HPO_4_, and 2.7-mM NaH_2_PO_4_, pH 7.2). Cells were rinsed throughout the process for periods of 10 minutes in fresh PBS, and nonspecific binding sites were blocked by incubation with 1% bovine serum albumin (BSA) and 0.2% Triton X-100 in PBS for 45 minutes at room temperature. Cells were incubated with polyclonal rabbit anti-P2Y_2_R, P2Y_4_R, and P2Y_6_R antibodies and goblet cell–specific CK7 antibody at 1:100 dilutions overnight at 4°C. Secondary antibodies conjugated to either Cy2 (1:100) or Cy3 (1:150) were incubated for 1.5 hours at room temperature. Incubation with control peptides and incubation in the absence of primary antibody served as negative controls.

### Measurement of Intracellular Calcium Concentration

The procedure was similar to that reported in previous articles.^21^^,^^27^^,^^28^ First-passage CGCs were cultured on 35-mm glass-bottom culture dishes and incubated at 37°C overnight. Cells were then incubated for 1 hour in the dark at room temperature with Krebs–Ringer bicarbonate (KRB)-HEPES supplemented with 0.5% BSA, 0.5-µM fura-2/AM, 8-µM pluronic acid F127, and 250-µM sulfinpyrazone. Prior to calcium measurements, cells were washed with KRB-HEPES containing 250-µM sulfinpyrazone. [Ca^2+^]*_i_* measurements following P2YR stimulation with nucleotides and specific agonists were conducted using a fluorescent ratio imaging system (In Cyt Im2; Intracellular Imaging, Cincinnati, OH, USA) with excitation wavelengths of 340 and 380 nm. The [Ca^2+^]*_i_* over time was displayed, and the change in peak [Ca^2+^]*_i_* was calculated by subtracting the average of the baseline value from the peak [Ca^2+^]*_i_* value.

### P2YR Depletion Using Short Interfering RNA

Short interfering RNA (siRNA) knockdown was conducted as described previously.^23^^,^^29^ siRNA specific to P2Y_2_R or P2Y_6_R or scrambled siRNA (ssRNA; Dharmacon, Lafayette, CO, USA) was added to RPMI 1640 medium at a final concentration of 100 nM. After 18 hours the media was replaced with fresh RPMI 1640 medium and incubated for 48 hours. Receptor depletion was confirmed with WB analysis using specific receptor antibodies as described in 2.6. ImageJ software (National Institutes of Health, Bethesda, MD, USA) was used to analyze blots.

### Measurement of High-Molecular-Weight Glycoprotein Secretion

The high-molecular-weight glycoprotein (HMWG) secretion was measured in the same manner as previously described.^23^ First-passage, cultured rat CGCs were grown in 24-well plates. Thereafter, the CGCs were serum starved for 2 hours before they were stimulated with agonists for 2 hours in serum-free RPMI 1640 medium supplemented with 0.5% BSA. An enzyme-linked lectin assay (ELLA) with UEA-I, which detects HMWGs such as MUC5AC, was exploited for goblet cell secretion measurements. The media were saved and analyzed for the amount of lectin-bound glycoproteins serving as indicator for goblet cell secretion.^30^ Secretion was expressed as fold increase over the baseline level, which was set to 1.

### Data Presentation and Statistical Analysis

Results are expressed as mean ± SEM, and data were analyzed by Student’s *t*-test for two group comparisons. All experiments were performed in triplicate on at least three animals. *P* < 0.05 was considered to indicate statistical significance. All graphs were generated and statistical analyses were performed using Prism 10 (GraphPad, Boston, MA, USA).

## Results

### Pyrimidinergic P2Y1-Like Nucleotide Receptors Were Detected With RT-PCR, WB, and IF Microscopy

**Figure 1.**
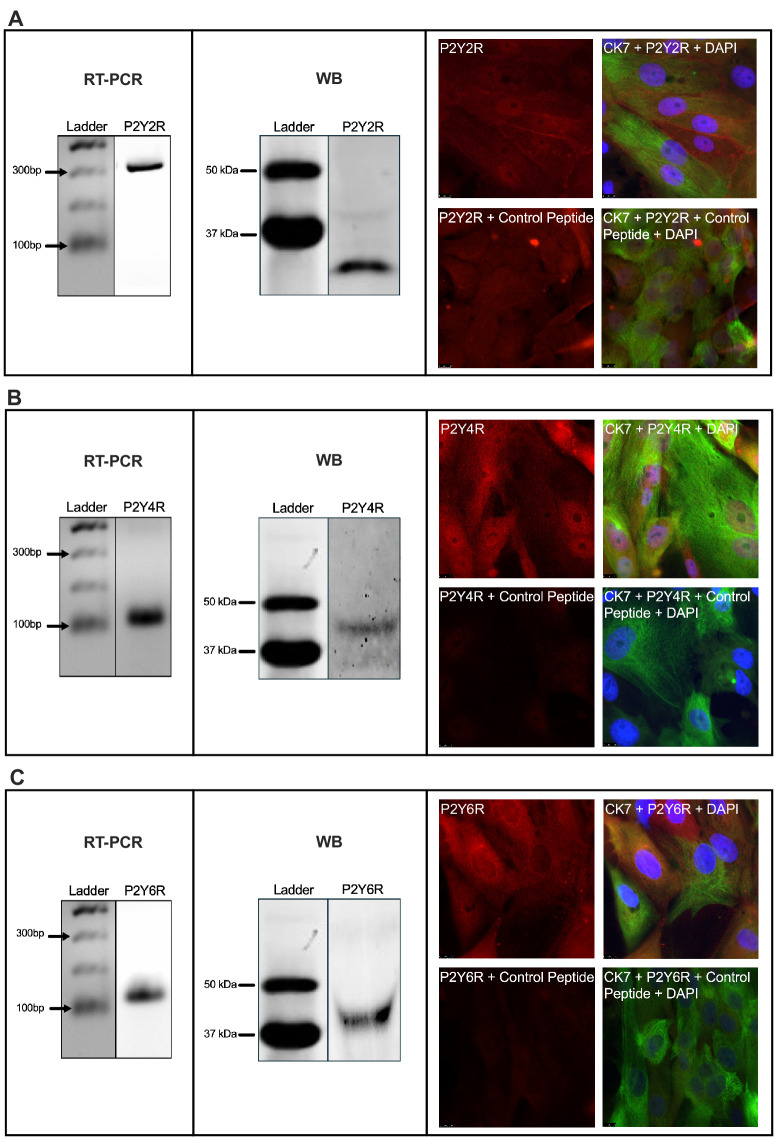
Rat conjunctival goblet cells contain P2Y_2_R, P2Y_4_R, and P2Y_6_R. (**A**–**C**) *Left panels* show that P2Y_2_R, P2Y_4_R, and P2Y_6_R mRNA was identified with RT-PCR in rat CGCs. The presence of P2YR mRNA was determined by RT-PCR on agarose gels. The *center panels* show that CGCs contain P2Y_2_R, P2Y_4_R, and P2Y_6_R proteins. The presence of P2YR proteins was determined by western blot. The *right panels* present immunofluorescence microscopy of P2Y_2_R, P2Y_4_R, and P2Y_6_R with respective peptide controls. Shown are micrographs at 1000× magnification of goblet cells stained with CK7 (*green*), DAPI (*blue*), and antibodies specifically targeting P2Y_2_R, P2Y_4_R, and P2Y_6_R (*red*). Control peptides were mixed with primary antibodies for P2YRs before IF microscopy. *N* = 3.

To our knowledge, in the conjunctiva P2Y_2_R is the only P2YR that has been extensively studied and published. To study the presence of UTP-activated P2Y1-like nucleotide receptor mRNA in cultured rat CGCs, total RNA was collected and mRNA was reverse transcribed using primers specific to each receptor subtype. mRNA for P2Y_2_R, P2Y_4_R, and P2Y_6_R was found as single bands of expected base-pair numbers by RT-PCR (Fig. 1, Supplementary Fig. S1).

WB analysis results for P2Y_2_R, P2Y_4_R, and P2Y_6_R in rat CGCs have not been previously published. Proteins extracted from CGCs from at least four rats were separated and visualized by WB using antibodies against specific P2YRs. Blots revealed bands reflecting the expected masses of each P2YR, except P2Y_2_R, which showed two bands (Fig. 1, Supplementary Fig. S2). Incubation with peptide control for P2Y_2_R antibody removed the lighter band, suggesting that this band with a mass below 37 kDa represented the antibody-bound isoform (Supplementary Fig. S2).

Next, CGCs were stained with the goblet cell marker anti-CK7 and co-labeled with antibodies specific to P2Y_2_R, P2Y_4_R, and P2Y_6_R. IF microscopy confirmed immunoreactivity of the three P2YRs in CGCs from the rats. Blocking peptides abolished staining with antibodies for all receptors, except for P2Y_2_R, suggesting an additional isoform of P2Y_2_R in cultured rat CGCs (Fig. 1). Staining patterns were different between P2Y_2_R incubated with control peptide missing nuclear and membrane immunoreactivity compared to P2Y_2_R antibody alone. Elimination of primary antibody decreased immunofluorescence for all P2YRs (Supplementary Fig. S3). To ensure the purity of cultures, cells were regularly double-labeled with the two goblet cell markers, UEA-1 and CK7, and visualized by IF microscopy (Supplementary Fig. S4).

### Uridine Triphosphate Increases [Ca^2+^]*_i_* in Cultured Rat CGCs

**Figure 2.**
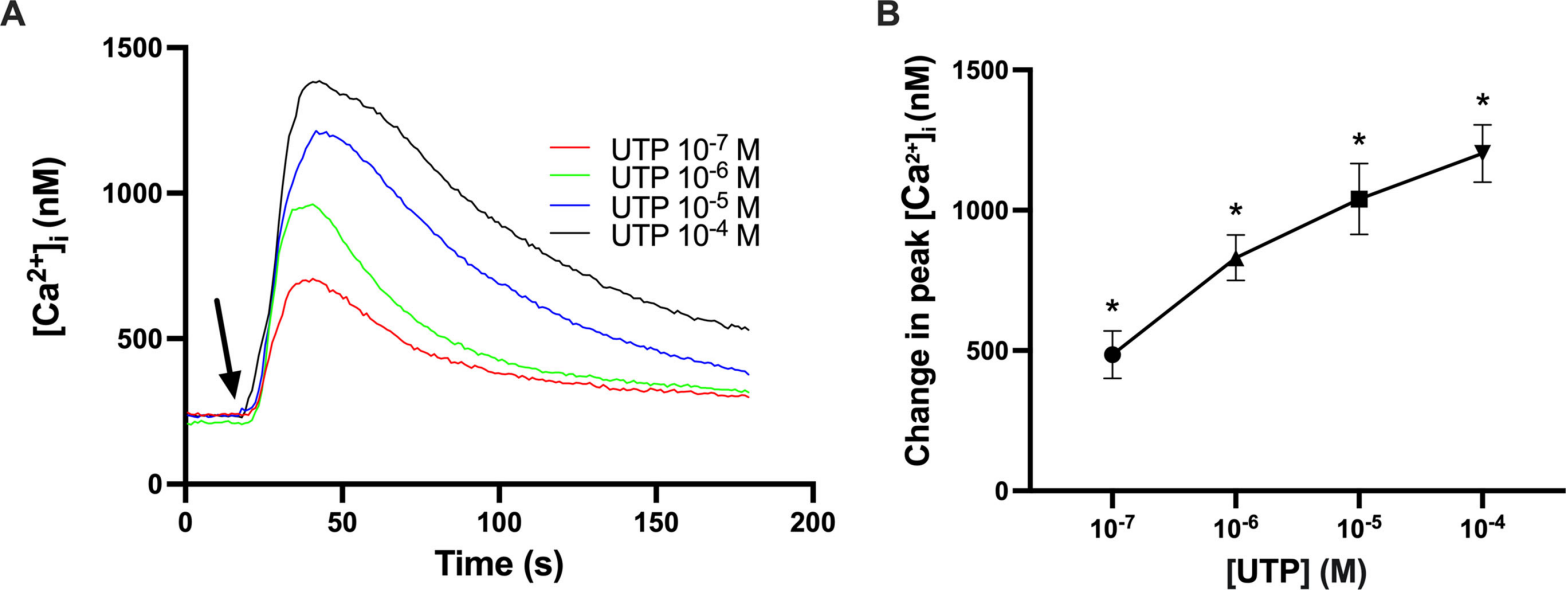
UTP stimulates an increase in [Ca^2+^]*_i_*. (**A**) The average [Ca^2+^]*_i_* levels for three animals over time after the addition of UTP (10^–7^–10^–4^ M) are shown. The *arrow* represents the addition of UTP. (**B**) Peak increase in [Ca^2+^]*_i_* above baseline for different concentrations of UTP. Data are presented as mean ± SEM. *Statistical significance above baseline.

UTP is an endogenous nucleotide known to activate P2Y_2_R, P2Y_4_R, and P2Y_6_R.^13^ To investigate the effects of UTP on changes in [Ca^2+^]*_i_* in rat CGCs, cells containing fura-2 were stimulated with UTP (10^–7^–10^–4^ M), and [Ca^2+^]*_i_* levels were measured (Fig. 2). UTP elicited a statistically significant, concentration-dependent increase in peak [Ca^2+^]*_i_* with values ranging from 486 ± 85 nM at UTP 10^–7^ M to 1202 ± 102 nM at UTP 10^–4^ M. These results suggest that one or more P2YRs from the class of pyrimidinergic P2Y1-like nucleotide receptors are functional in rat CGCs.

### Specific Agonists Targeting P2Y_2_R, P2Y_4_R, and P2Y_6_R All Increase [Ca^2+^]*_i_* in Cultured Rat CGCs

**Figure 3.**
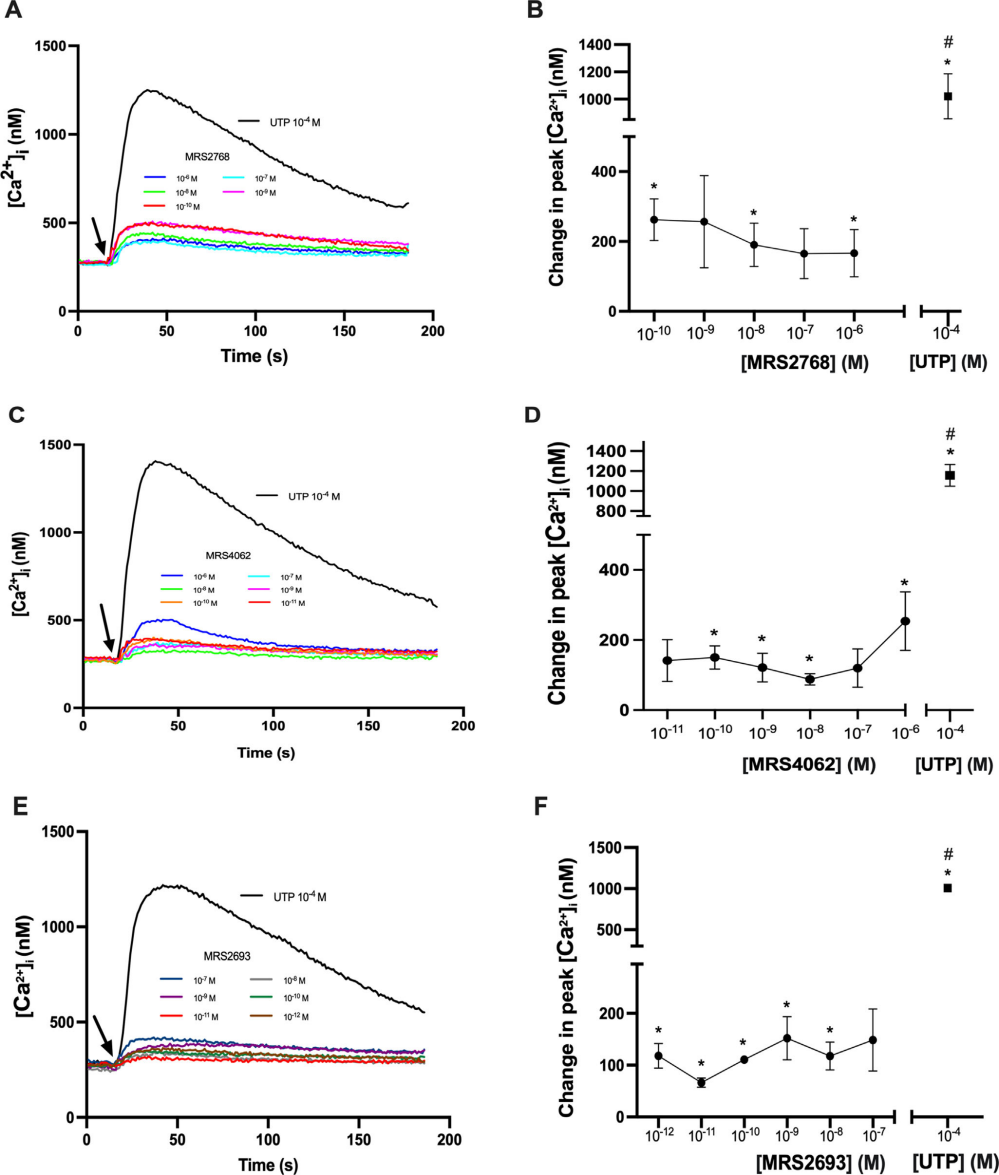
The P2YR-specific agonists MRS 2768, MRS 4062, and MRS 2693 increase [Ca^2+^]*_i_* in rat conjunctival goblet cells. (**A**, **B**) The effect of MRS 2768 (P2Y_2_R agonist) on [Ca^2+^]*_i_*. The average [Ca^2+^]*_i_* levels for four rats over time are shown in **A**, and **B** shows the peak increase in [Ca^2+^]*_i_* above baseline for the four rats, with *closed circles* representing increasing concentrations of MRS 2768, and *closed square* representing UTP 10^–4^ M. (**C**, **D**) The effect of MRS 4062 (P2Y_4_R agonist) on [Ca^2+^]*_i_*. The average [Ca^2+^]*_i_* levels for three rats over time are shown in **C**, and **D** shows the peak increase in [Ca^2+^]*_i_* above baseline for the three rats, with *closed circles* representing increasing concentrations of MRS 4062, and closed square representing UTP 10^–4^ M. (**E**, **F**) The effect of MRS 2693 (P2Y_6_R agonist) on [Ca^2+^]*_i_*. The average [Ca^2+^]*_i_* levels for the three rats over time are shown in **E**, and **F** shows the peak increase in [Ca^2+^]*_i_* above baseline for the three rats, with *closed circles* representing increasing concentrations of MRS 2693, and closed square representing UTP 10^–4^ M. *Arrows* represent the addition of MRS 2768 (**A**), MRS 4062 (**C**), MRS 2693 (**E**), or UTP (**A**, **C**, **E**). Data are presented as mean ± SEM. *Statistical significance above baseline; #statistical significance from specific agonists at all concentrations.

To determine the effects of either of the three P2YRs activated by UTP, CGCs were stimulated with agonists specific to each P2YR and [Ca^2+^]*_i_* measured. MRS 2768, an agonist specific to P2Y_2_R, was applied to fura-2 containing CGCs at concentrations ranging from 10^–10^ to 10^–6^ M.^31^ All concentrations except MRS 2768 10^–9^ M (*P* = 0.0733) and 10^–7^ M (*P* = 0.051) elicited statistically significant increases in peak [Ca^2+^]*_i_*. The human P2Y_4_R-specific agonist MRS 4062 stimulated statistically significant increases in peak [Ca^2+^]*_i_* at all concentrations except for 10^–11^ M and 10^–7^ M (*P* = 0.07 and *P* = 0.08, respectively). Similar to MRS 2768 and MRS 4062, the human P2Y_6_R-specific agonist MRS 2693 increased [Ca^2+^]*_i_* significantly from the baseline level at all but one concentration (10^–7^ M; *P* = 0.0657) (Fig. 3). Altogether, results from the application of specific agonists to P2YRs activated by UTP induced lower peak [Ca^2+^]*_i_* responses than UTP 10^–4^ M alone, indicating coactivation of several receptors by UTP.

### P2Y_6_R- but not P2Y_14_R-Specific Agonist Increases [Ca^2+^]*_i_* in Rat CGCs

**Figure 4.**
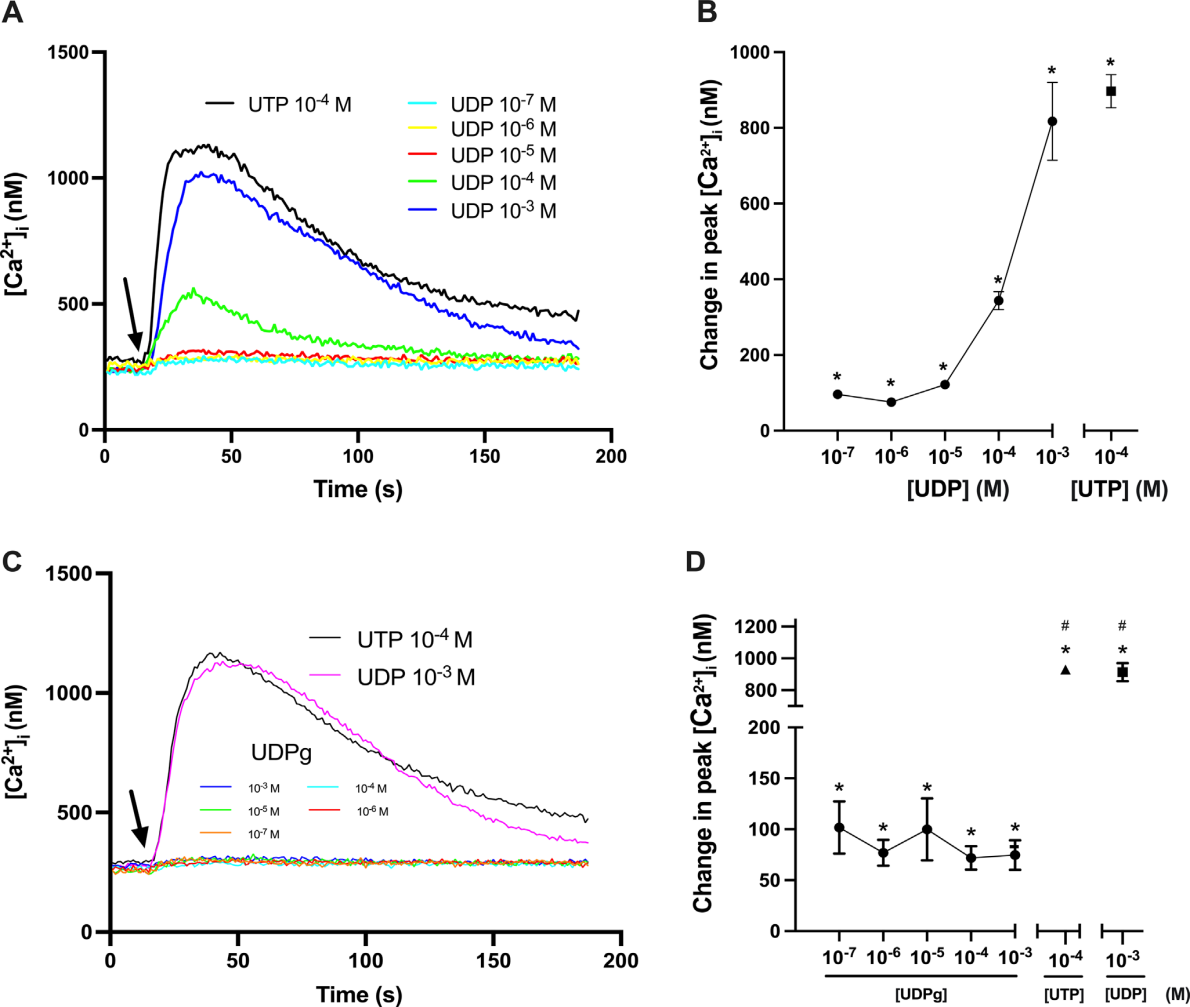
UDP but not UDP–glucose (UDPg), increases [Ca^2+^]*_i_* in rat conjunctival goblet cells. (**A**, **B**) The effect of UDP (P2Y_6_R and P2Y_14_R agonist) on [Ca^2+^]*_i_*. The average [Ca^2+^]*_i_* levels for three rats over time are shown in **A**, and **B** shows the peak increase in [Ca^2+^]*_i_* from baseline for the three rats, with *closed circles* representing increasing concentrations of UDP, and a *closed square* representing UTP 10^–4^ M. (**C**, **D**) The effect of UDPg (P2Y_14_R agonist) on [Ca^2+^]*_i_*. The average [Ca^2+^]*_i_* levels for three rats over time are shown in **C**, and **D** shows the peak increase in [Ca^2+^]*_i_* from baseline for the three rats, with *closed circles* representing increasing molar concentrations of UDP-glucose; *closed* *triangle*, UTP 10^–4^ M; and *closed square*, UDP 10^–3^ M. *Arrows* represent the addition of UDP (**A**, **C**), UDPg (**C**), or UTP (**A**, **C**). *Statistical significance above baseline; #statistical significance from UDPg at all concentrations. Data are presented as mean ± SEM. *N* = 3.

In contrast to the partial agonist UTP, UDP is a full agonist of P2Y_6_R.^13^ This pyrimidine nucleoside diphosphate is selective for P2Y_6_R and P2Y_14_R.^13^ UDP–glucose, however, is an endogenous ligand to P2Y_14_R but not P2Y_6_R.^13^ To investigate which receptor stimulates changes in [Ca^2+^]*_i_* in rat CGCs, cultured cells were loaded with fura-2 and stimulated with either UDP (10^–7^–10^–3^ M) or UDP–glucose (10^–7^–10^–3^ M), and [Ca^2+^]*_i_* levels were measured (Fig. 4). UDP increased [Ca^2+^]*_i_* in a dose-dependent manner from 96 ± 3 nM at UDP 10^–7^ M to 817 ± 103 nM at UDP 10^–3^ M (Figs. 4A, 4B). In the same experiment, UTP 10^–4^ M increased [Ca^2+^]*_i_* to 896 ± 44 nM from the baseline level (Fig. 4B). Change in peak [Ca^2+^]*_i_* after stimulation with UDP–glucose (10^–7^–10^–3^ M) was statistically significant but showed no obvious dose dependency and ranged from only 102 ± 26 nM at UDP–glucose 10^–7^ M to 75 ± 15 nM at 10^–3^ M (Figs. 4C, 4D). These results suggest that P2Y_6_R, but not P2Y_14_R, acts by increasing [Ca^2+^]*_i_* in rat CGCs. In addition, UDP and UTP stimulated similar increases in peak [Ca^2+^]*_i_*, indicating that P2Y_6_R is functional among UTP-activated P2YRs. Also, UDP, like UTP, activates receptors other than P2Y_6_R to increase [Ca^2+^]*_i_*.

### Action of P2Y_2_R and P2Y_6_R siRNA on Agonist-Induced Peak [Ca^2+^]*_i_*

**Figure 5.**
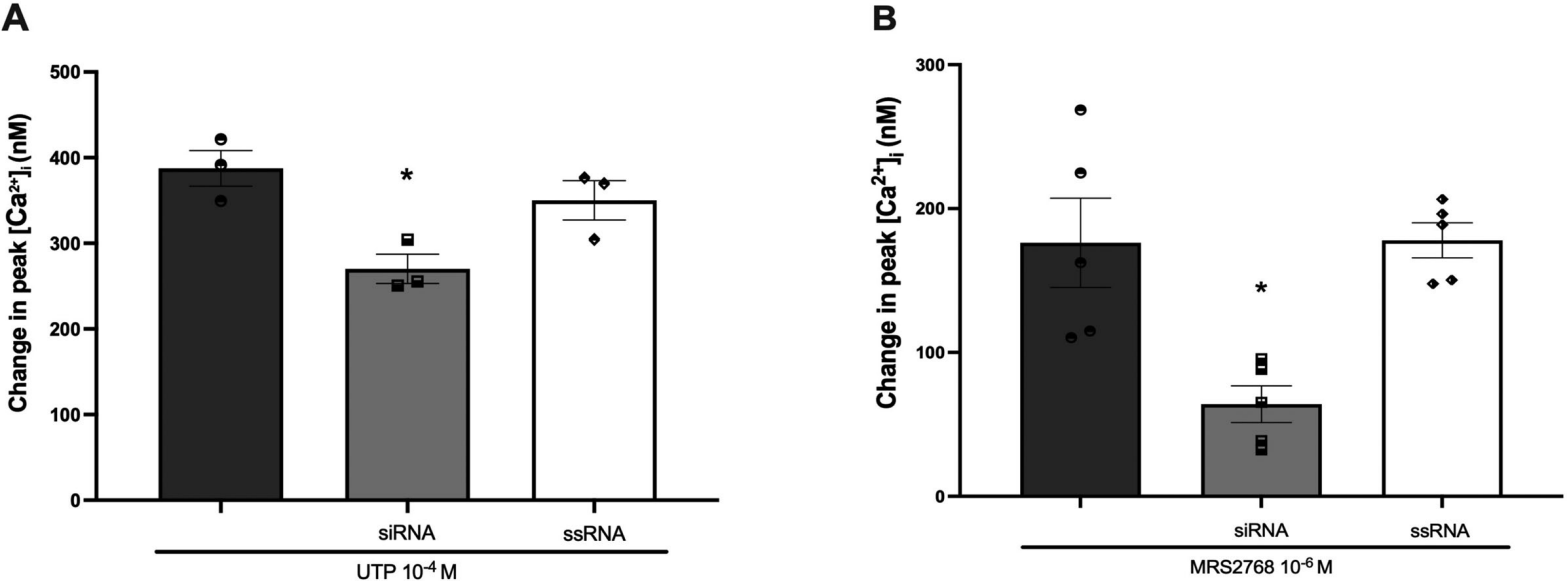
P2Y_2_R knockdown. (**A**, **B**) The effect of knockdown of P2Y_2_R expression on UTP- and MRS 2768-induced peak [Ca^2+^]*_i_*. Rat CGCs were treated with 100 nM P2Y_2_R siRNA (*light* *gray* *bars*), scrambled siRNA (ssRNA) (*white bars*), or no siRNA (*dark* *gray* *bars*) before the addition of UTP (10^−4^ M) (**A**) or MRS 2768 (10^−6^ M) (**B**). Data are presented as mean ± SEM. *Statistically significant difference from agonist alone. *N* = 3 for **A** and *N* = 5 for **B**.

To investigate if the peak [Ca^2+^]*_i_* increase in response to UTP and the P2Y_2_R agonist MRS 2768 addition was in part due to P2Y_2_R activation, cultured rat CGCs were treated with 100-nM P2Y_2_R siRNA and stimulated with UTP (10^−4^ M) (Fig. 5A) or MRS 2768 (10^−6^ M) (Fig. 5B) before [Ca^2+^]*_i_* measurements. P2Y_2_R expression after siRNA and ssRNA treatments was calculated by WB analysis (Supplementary Figs. S5A, S5B) and was found to be decreased by 54% and increased by 10%, respectively, compared to non-treated CGCs. Peak [Ca^2+^]*_i_* significantly decreased from 388 ± 21 nM and 176 ± 31 nM for non-treated CGCs stimulated with UTP 10^−4^ M and MRS 2768 10^−6^ M, respectively, to 270 ± 17 nM and 64 ± 13 nM, respectively, for CGCs treated with siRNA. The negative control, ssRNA, did not significantly reduce UTP or MRS 2768 peak [Ca^2+^]*_i_*. In conclusion, activity of P2Y_2_R in CGCs is supported by siRNA experiments that show a reduction in peak [Ca^2+^]*_i_* in response to UTP and MRS 2768 after P2Y_2_R depletion.

**Figure 6.**
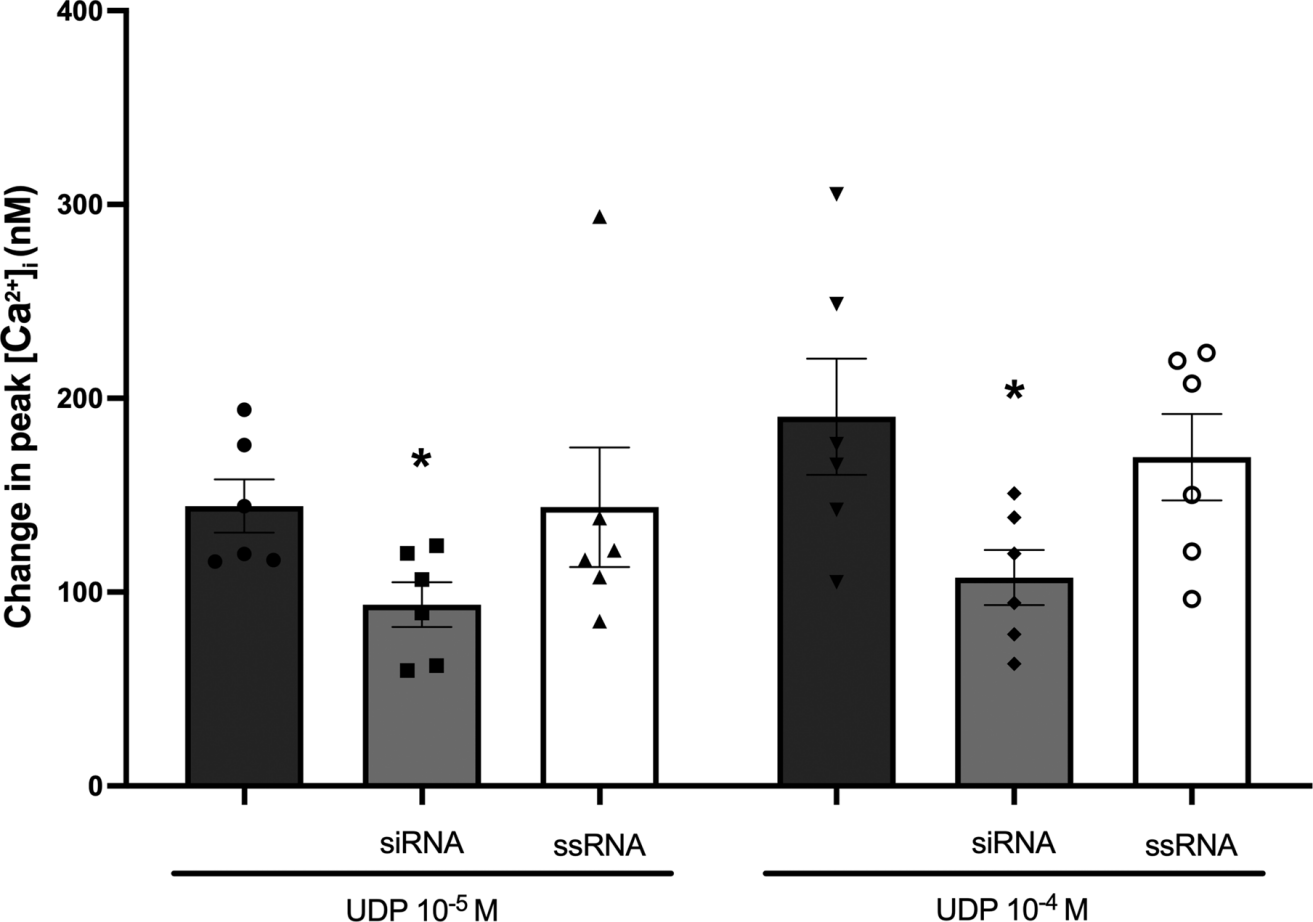
P2Y_6_R knockdown. CGCs were treated with 100-nM P2Y_6_R siRNA, scrambled siRNA (ssRNA), or no siRNA before the addition of UDP (10^−5^ or 10^−4^ M). The *dark* *gray* *bars* represent UDP alone, *light* *gray* *bars* represent UDP after siRNA treatment, and *white bars* represent UDP after ssRNA treatment. All data are presented as mean ± SEM. *Statistical significance from agonist alone. *N* = 6.

To determine if UDP activates P2Y_6_R to increase [Ca^2+^]*_i_*, cultured rat CGCs were treated with 100-nM siRNA to knock down P2Y_6_R expression following UDP addition (Fig. 6); ssRNA was used as the negative control. The percentage of P2Y_6_R expression after siRNA treatment was knocked down by 57% compared to non-treated cells (Supplementary Figs. S6A, and S6B). When ssRNA was used, P2Y_2_R expression was up by 1% compared to non-treated cells. As shown in Figure 6, peak [Ca^2+^]*_i_* was reduced from 144 ± 14 nM at UDP 10^−5^ M and 191 ± 30 nM at UDP 10^−4^ M to 94 ± 11 nM and 108 ± 14 nM, respectively, for non-transfected cells and cells transfected with siRNA. ssRNA treatment of CGCs did not significantly alter the UDP-induced peak [Ca^2+^]*_i_*. In summary, a functional P2Y_6_R in cultured rat CGCs is supported by a reduction in UDP-stimulated peak [Ca^2+^]*_i_* in CGCs depleted of P2Y_6_R protein.

### Induction of Mucin Secretion by Pyrimidinergic P2Y1-Like Receptor Agonists

**Figure 7.**
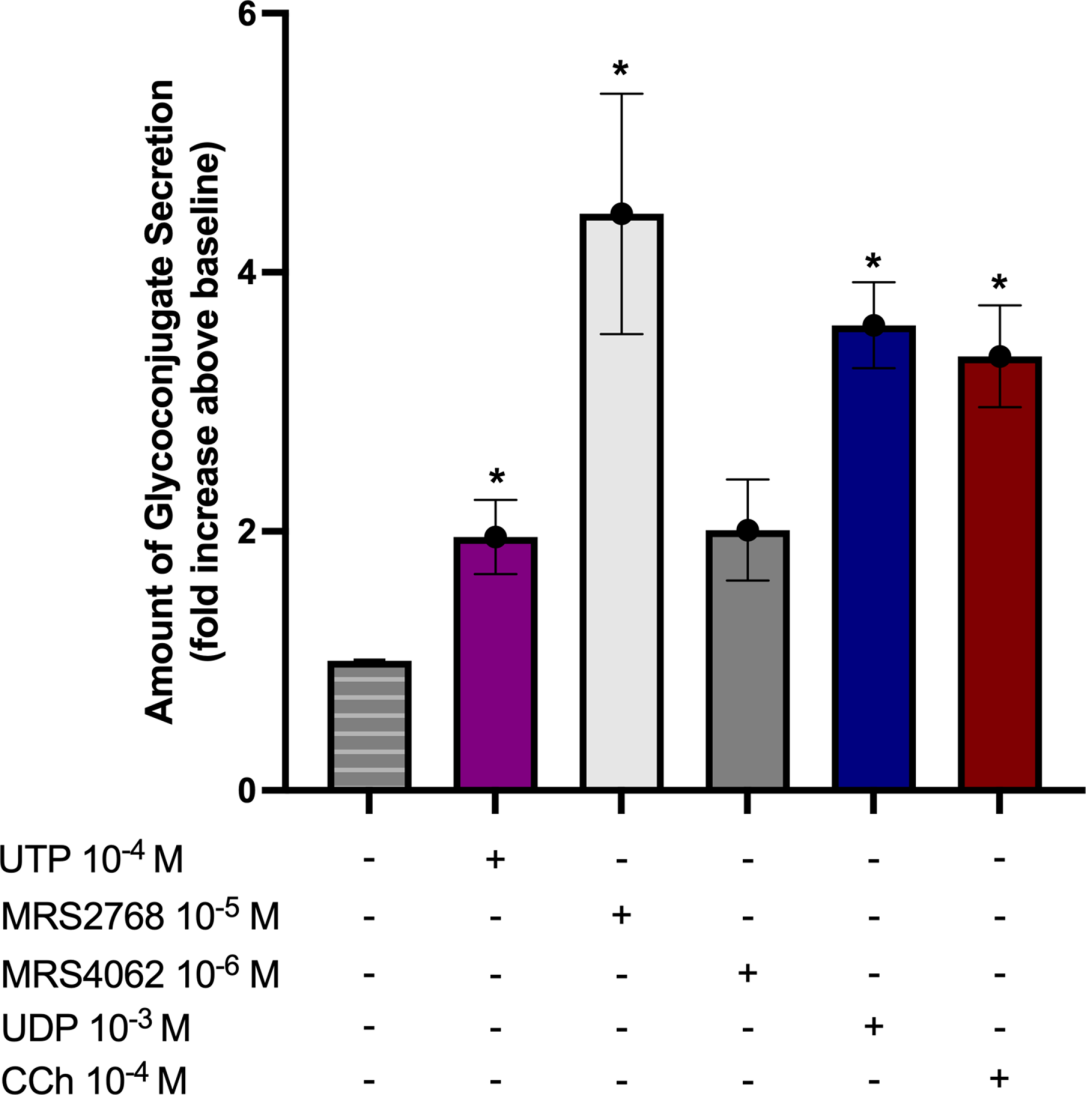
P2Y1-like nucleotide receptor activation induces mucin secretion from rat CGCs. Rat conjunctival goblet cells were stimulated with agonists for 2 hours before high-molecular-weight protein secretion measurements. *Striped bar*, baseline (negative control); *purple bar*, UTP 10^–4^ M; *white bar*, MRS 2768 (P2Y_2_R agonist) 10^–5^ M; *gray* *bar*, MRS 4062 (P2Y_4_R agonist) 10^–6^ M; *blue bar*, UDP (P2Y_6_R and P2Y_14_R agonist) 10^–3^ M; *red bar*, positive control carbachol 10^–4^ M. Data are mean ± SEM. *Statistical significance from baseline set to 1.0. *N* = 4.

To investigate if UTP and agonists specific to P2Y_2_R, P2Y_4_R, and P2Y_6_R stimulate secretion in cultured rat CGCs, cultured cells were stimulated with agonists for 2 hours and HMWG secretion measured. Concentrations of agonists were chosen based on reported half maximal effective concentration (EC_50_) values in the literature and [Ca^2+^]*_i_* measurements.^13^ UTP (P2Y_2_R, P2Y_4_R, and P2Y_6_R selective; 10^–4^ M), MRS 2768 (P2Y_2_R specific; 10^–5^ M), MRS 4062 (P2Y_4_R specific; 10^–6^ M), and UDP (P2Y_6_R specific; 10^–3^ M) stimulated HMWG secretion to 1.9 ± 0.2-, 4.4 ± 0.9-, 2.0 ± 0.3-, and 3.5 ± 0.3-fold over baseline levels (Fig. 7). Carbachol (10^–4^ M), a muscarinic agonist shown to stimulate HMWG secretion from rat CGCs, was used as the positive control. Although not statistically significant (*P* = 0.06), P2Y_4_R stimulation led to a twofold increase in HMWG secretion from the baseline level. These results indicate that activation of both P2Y_2_R and P2Y_6_R induces mucin secretion in rat CGCs similar to that of muscarinic activation.

## Discussion

In this study, we demonstrated the presence of a pyrimidinergic P2Y1-like nucleotide subgroup of P2Y receptors. P2Y_2_R, P2Y_4_R, and P2Y_6_R were investigated at both the mRNA and protein levels in rat CGCs through RT-PCR, WB, and IF microscopy. Using endogenous ligands as agonists for these receptors, we showed that UTP (P2Y_2_R, P2Y_4_R, and P2Y_6_R agonist) and UDP (P2Y_6_R and P2Y_14_R agonist), but not UDP–glucose (P2Y_14_R agonist) were powerful stimulators of [Ca^2+^]*_i_* in rat CGCs. By applying specific P2YR agonists, we confirmed activity for P2Y_2_R, P2Y_4_R, and P2Y_6_R in rat CGCs. Surprisingly, specific agonists had limited effects on [Ca^2+^]*_i_* compared to the endogenous pyrimidine nucleotides UTP and UDP. These endogenous ligands, such as the purine nucleotide adenosine triphosphate (ATP), likely target other receptors in addition to P2YRs.^14^^,^^32^

We eliminated a role for shear stress in causing the peak [Ca^2+^]*_i_* levels, as vehicle addition increased [Ca^2+^]*_i_* to 42 ± 15 nM (data not shown), and most of the calculated peak [Ca^2+^]*_i_* levels after specific agonist addition were substantially higher. Hence, shear stress force on CGCs, combined with compound additions and subsequent nucleotide release, is likely to have a minimal effect on [Ca^2+^]*_i_* increases in most experiments.

The profile of P2Y receptor stimulation of secretion was different from that of [Ca^2+^]*_i_*. The specific agonist for P2Y_2_R, MRS 2768, was more effective at stimulating secretion than the endogenous analogs UTP and UDP. This finding suggests that an increase in [Ca^2+^]*_i_* might not directly lead to an increase in CGC secretion. Because all HMWG secretion measurements were expressed as fold changes over the baseline level, and thereby normalized, differences resulting from early inadvertent secretion by CGC handling were avoided.

In terms of UTP-stimulated increases in HMWG secretion, results from our study are consistent with a previous study on HMWG secretion in rabbit nictitans and human bulbar conjunctiva.^18^ The authors found that ATP, ADP, and UTP led to a large increase in mucin release and ascribed this finding to a P2Y_2_R agonist profile. In the same study, however, neither the putative P2 receptor antagonist suramin nor reactive blue significantly influenced mucin release upon ATP addition to rabbit nictitans.^18^ In our study, application of the P2Y_2_R agonist MRS 2768 led to a large increase in HMWG secretion, greater than UTP and UDP. Unlike in previous studies, in our study the effect of uridine nucleotides and specific P2YR agonists on increasing the intracellular signaling molecule Ca^2+^ was determined. In contrast to secretion, the nucleotides UTP and UDP increased [Ca^2+^]*_i_* to a greater extent than the P2Y_2_R-specific agonist MRS 2768. P2Y_2_R mainly couples to G_q_; however, some studies suggest P2YR stimulation of additional signaling pathways that involve second messengers other than inositol-3-phosphate.^33^ This may indicate additional [Ca^2+^]*_i_* independent mechanisms for mucin secretion upon P2Y_2_R stimulation in rat CGCs.^34^ In addition, the nucleotides appear to have different effects than the specific agonists.

One reason why the effects of nucleotides may differ on [Ca^2+^]*_i_* and secretion compared with specific synthetic agonists is that nucleotides can be released to the extracellular environment (e.g., the ocular surface^35^) by exocytosis, shear stress, inflammation, mechanical stimulation, or receptor activation.^36^^,^^37^ Subsequently, the nucleotides are exposed to extracellular enzymes, ectonucleotidases, that can hydrolyze the triphosphates into diphosphates, monophosphates, and single nucleosides. In the extracellular environment, these metabolites can activate the membrane-bound P1 and P2 (P2X and P2Y) receptors as they are metabolized, whereas the specific P2YR agonists are not metabolized and only activate one type of receptor.

Despite recent advancements in the availability of ligands for the investigation of P2YRs, various complexities continue to impede research progress in the field.^38^ In the present study, specific agonists were carefully selected based on features such as potency, selectivity, and commercial availability. However, pEC_50_ values for G protein–coupled receptor agonists can differ significantly between test systems and receptor expression, making it difficult to extract causal relationships.^39^ Therefore, several concentrations of each substance were investigated, and agonists were aliquoted immediately in specific solutions and stored at –70°C to prevent degradation. The protein concentrations of each P2YR were not quantified in this study. It is reported that species homologs of P2YR subtypes have a high degree of homology, and highly conserved receptor regions are most likely involved in ligand binding.^40^ Still, differences in receptor action among species are observed. Although ligands have shown promising results for human P2YRs, rat P2YRs and human P2YRs are not entirely similar. For example, in humans ATP functions as an antagonist to P2Y_4_R, whereas in rats ATP exhibits agonistic activity on the same receptor, suggesting differences in agonist binding sites among species.^41^^,^^42^ Similarly, ATP- and UTP-sensitive P2Y_11_R has been described as being missing from the rat genome, reflecting species differences; therefore, no effort was made to elucidate this subtype in the present study.^12^^,^^40^

MRS 2768, which is specific for P2Y_2_R and displays no affinity for P2Y_4_R or P2Y_6_R, is reported to increase [Ca^2+^]*_i_* in astrocytes from the rat hypothalamus.^31^ In the present study, MRS 2768 increased [Ca^2+^]*_i_* and induced the highest mucin secretory response in cultured rat CGCs. Furthermore, depletion of P2Y_2_Rs using siRNA decreased peak intracellular [Ca^2+^]*_i_*. INS365 (diquafosol) is a synthetic organic compound that stimulates human P2Y_2_R and was developed for the treatment of patients with DED in Asian countries.^43^ MRS 4062 (P2Y_4_R agonist) increased [Ca^2+^]*_i_* in this study and is reportedly an agonist with superior selectivity and potency for P2Y_4_R. This agonist is twice as potent as UTP and has 27- and 32-fold greater selectivity for P2Y_4_R compared to P2Y_2_R and P2Y_6_R, respectively.^44^ The P2Y_4_R agonist did not, however, induce significant mucin secretion in our rat CGCs, although it was not far from statistical significance (*P* = 0.06). MRS 4062 has been utilized for the identification and characterization of P2Y_4_R in rat cardiac fibroblasts.^45^ Together with P2Y_2_R, P2Y_4_R is responsible for nucleotide-promoted Cl^–^ responses in the upper intestinal tract, but P2Y_4_R seems to be the only receptor responsible for this kind of Cl^–^ secretion in the colon.^40^^,^^46^^,^^47^ As other studies have shown abundant P2Y_4_R expression and secretory function in epithelia, it is possible that P2Y_4_R plays important roles in electrolyte and water secretion in conjunctival epithelial cells that were not measured in the present study.^48^

The last specific agonist we used in this study was one for P2Y_6_R. In our study, the P2Y_6_R agonist MRS 2693 slightly increased [Ca^2+^]*_i_*, but its effect on mucin secretion in cultured rat CGCs was not measured. UDP that activates P2Y_6_R stimulated both an increase in [Ca^2+^]*_i_* and secretion from the CGCs. Furthermore, peak [Ca^2+^]*_i_* was reduced after P2Y_6_R depletion. Thus, P2Y_6_R likely plays an important role in CGC function, and the nucleotide is more effective than the specific agonist; however, extracellular enzymatic conversion of UDP to UTP, and conversely UTP to UDP, can potentially partially explain nucleotide activation of several pyrimidinergic P2YRs.^14^ In humans, MRS 2693 displays no activity against P2YRs other than P2Y_6_R and is equipotent as UDP on P2Y_6_R.^44^^,^^49^ We have found only one study applying MRS 2693 in rats, and the authors found its effect to be equivalent to that of UDP on microglia-dependent neuronal loss, suggesting a substantial effect on P2Y_6_R also in rats.^50^ This finding is in contrast to the findings of the present study regarding rat CGCs.

Like P2Y_2_R and P2Y_4_R, P2Y_6_R promotes ion transport in epithelial cells.^51^^,^^52^ This could occur in CGCs but remains to be tested. P2Y_6_R is widely expressed in different tissues, but difficulties in separating its actions from those of P2Y_2_R and P2Y_4_R has hindered research.^40^ Nonetheless, recent evidence suggests a compelling role for P2Y_6_R in glaucoma treatment.^53^^,^^54^ This receptor also reduces inflammation via suppression of NF-κB activation.^55^^,^^56^ Activation of P2Y_6_R may therefore represent a compelling treatment target for both DED and glaucoma in elderly patients, where these conditions are often seen combined.^57^ The treatment of glaucoma using topical medications has been shown to increase the risk of having DED,^58^ but this could either be an effect of the preservative on the ocular surface or an effect of altering P2Y_6_R activity in CGCs by its agonist.

We conclude that P2Y_2_R, P2Y_4_R, and P2Y_6_R are present in rat CGCs and that UTP and UDP, as well as a specific P2Y2R agonist, stimulate mucin secretion that can contribute to tear film stability. Our findings represent important knowledge for the development of novel compounds to treat and prevent ocular surface disease.

## Supplementary Material

Supplement 1
