# Supplementary material for: Pyrimidinergic P2Y1-Like Nucleotide Receptors Are Functional in Rat Conjunctival Goblet Cells
Source: Invest Ophthalmol Vis Sci. 2025 Jan 21;66(1):46. doi: 10.1167/iovs.66.1.46 (PMC11753474; doi:10.1167/iovs.66.1.46)
Supplement: Supplement 1 [file iovs-66-1-46_s001.pdf]

Pyrimidinergic P2Y1-like Nucleotide Receptors are Functional in Rat Conjunctival Goblet Cells

Supplementary material

Supplemental Table 1. Primers for RT-PCR

Receptor	Primer Sequences 5´-3´	Annealing Temp (°C)	Fragment Size (bp)	Accession No.*
P2Y$_2$	CTGCCAGGCACCCGTGCTCTACTT<br>CTGAGGTCAAGTGATCGGAAGGAG	60	339	XM_039109<br>599
P2Y$_4$	CATGAGGAAAGCATCAGCAG<br>CCCTTCATATCCAGCAGCAG	60	100	NM_031680
P2Y$_6$	GGCAGTGTCCTTTCTATGAGG<br>GGCAATGAACAAAGTCCACA	60	135	NM_057124
β-Actin	AGGCCAACCGTGAAAAGATG<br>ACCAGAGGCATACAGGGACAA	60	101	NM_031144

**GenBank, National Institutes of Health, Bethesda, MD; http://www.ncbi.nlm.nih.gov/genbank*

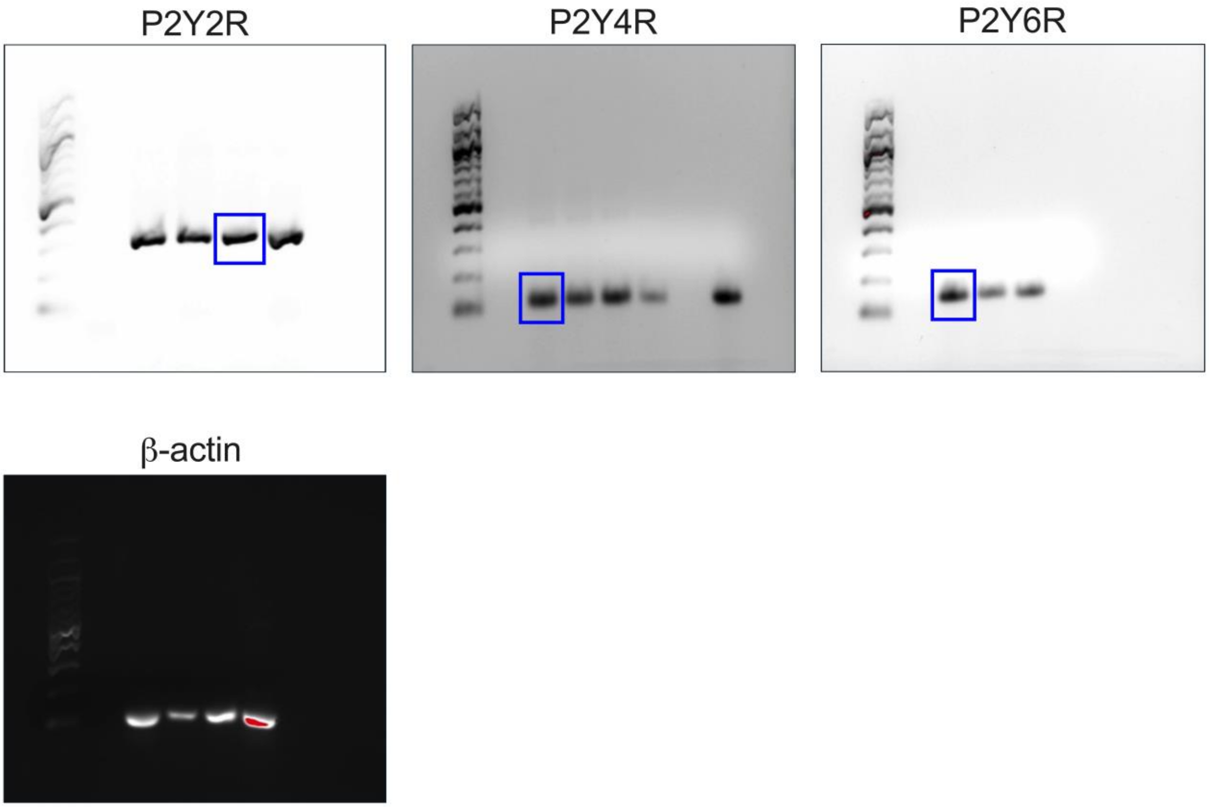

Supplemental Fig. 1 Individual gels for RT-PCR. Single bands were detected for all studied P2YRs and β-actin. Upper panel from left to right: P2Y$_2$R (339 bp); P2Y$_4$R (100 bp); P2Y$_6$R (135 bp). Lower panel:

β-actin (101 bp). Blue boxes depict bands in Fig. 1 in main article. RT-PCR on β-actin mRNA was done for corresponding rat samples as a positive control. N=3-5

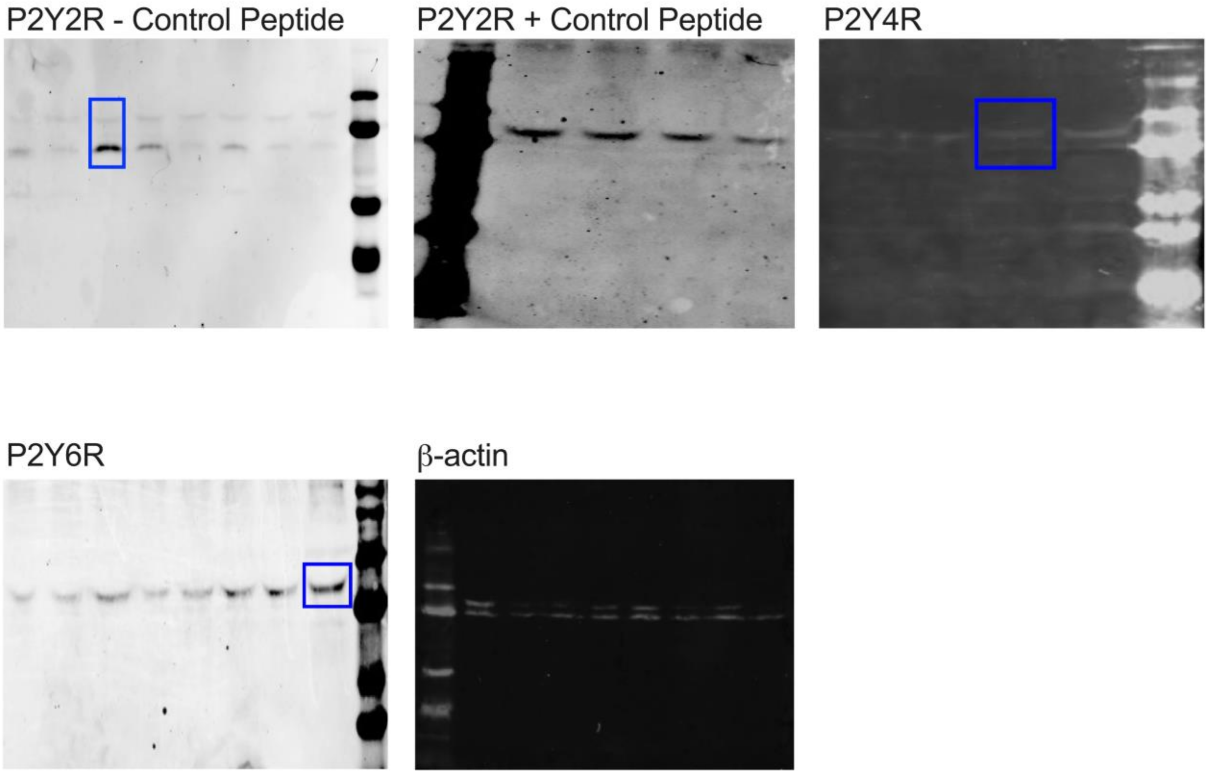

Supplemental Fig. 2 Individual Western blots. Bands were detected for all investigated P2YRs. Upper panel from left to right: $P2Y_2R$ (42.3 kDa) without control peptide; $P2Y_2R$ with control peptide; $P2Y_4R$ (40.894 kDa). Lower panel from left to right: $P2Y_6R$ (36.721 kDa); β-actin (42 kD). Blue boxes depict bands in Fig. 1 in main article. Western blotting on β-actin was done for corresponding rat samples as a positive control. N=4-8

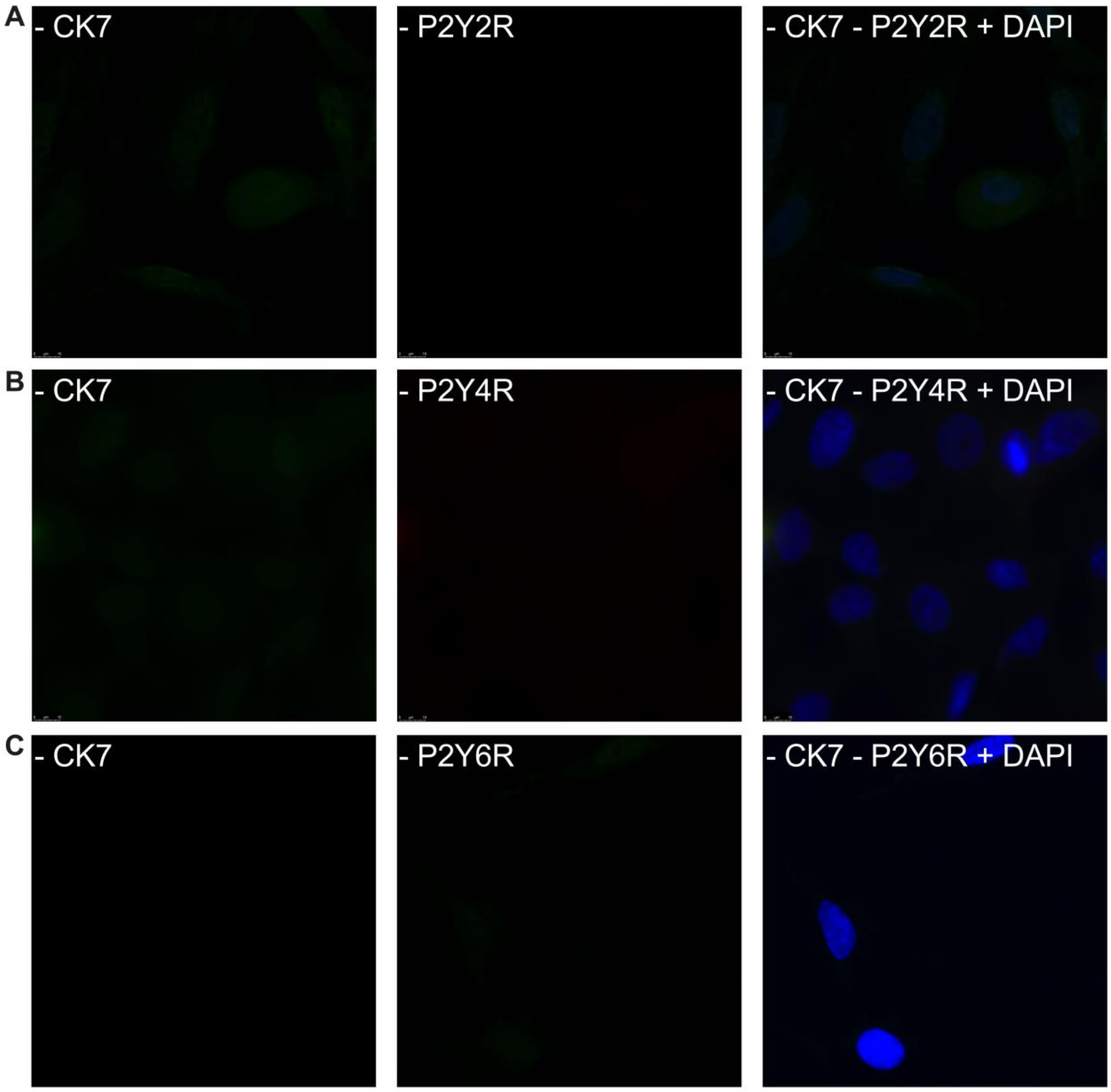

Supplemental Fig. 3 Immunofluorescence microscopy without primary antibodies. Negative control for (**A**) $P2Y_2R$, (**B**) $P2Y_4R$, (**C**) $P2Y_6R$. CGCs were incubated with secondary antibodies, but not primary antibodies against P2YRs and CK7. Green= CK7, red= P2YRs, and blue= DAPI. Images are representative for at least three rats. Magnification, X1000

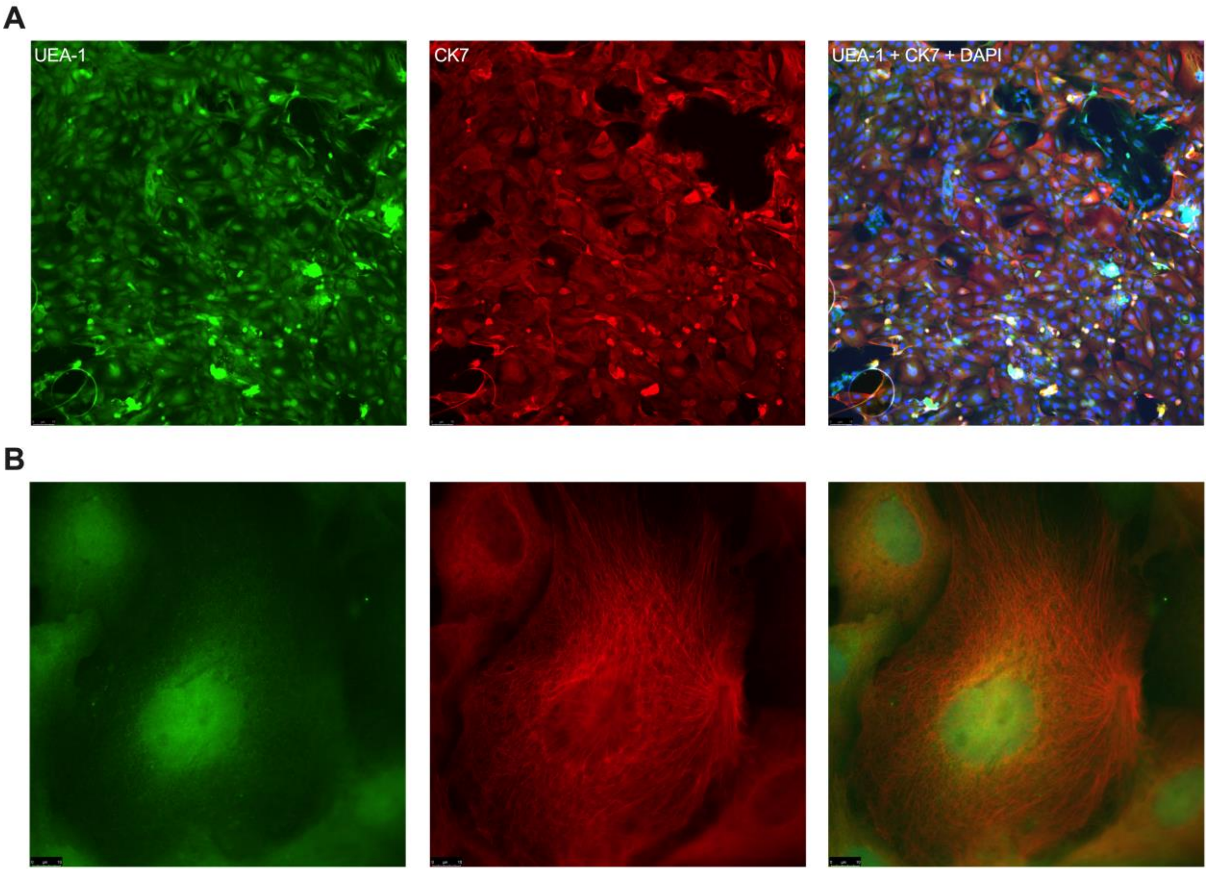

Supplemental Fig. 4 Immunofluorescence microscopy confirming goblet cell identity. Green= UEA-1, red= CK7, blue= DAPI. (**A**) magnification, 100X; (**B**) magnification, 1000X. N=3

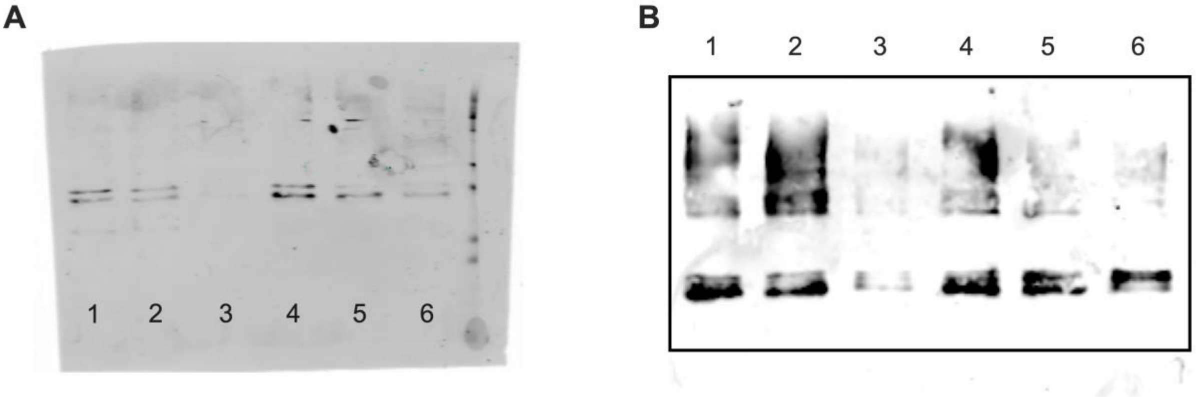

Supplemental Fig. 5 Western blotting analysis of P2Y$_2$R siRNA-treated CGCs. (**A**) shows the Western blot of P2Y$_2$ protein after siRNA treatment (lanes 1 and 2), ssRNA treatment (lanes 3 and 4) or no treatment (lanes 5 and 6) of cultured rat CGCs. (**B**) presents Western blot for β-actin with the same treatments of CGCs as (**A**)

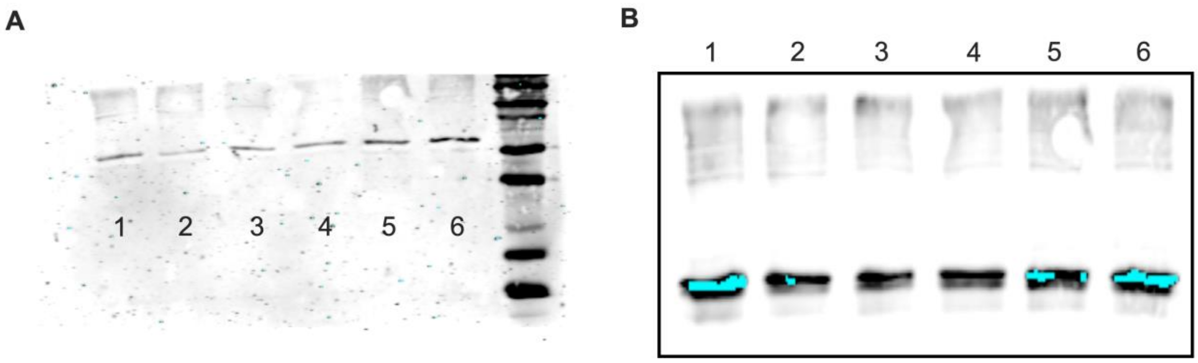

Supplemental Fig. 6 Western blotting analysis of P2Y$_6$R siRNA-treated CGCs. (**A**) illustrates the Western blot of P2Y$_6$R protein after no treatment (lanes 5 and 6), ssRNA treatment (lanes 3 and 4) or P2Y$_6$R siRNA treatment (lanes 1 and 2). (**B**) presents the Western blot for β-actin with the same treatments of CGCs as (**A**)
